# Protein intake, physical activity, and skeletal muscle outcomes in middle-aged adults: an observational study from the US

**DOI:** 10.1017/jns.2026.10124

**Published:** 2026-07-17

**Authors:** Konstantinos Prokopidis, Brendon Stubbs, Nicola Veronese, Stefano Cacciatore, Paolo Piaggi, Carla M. Prado, John A. Batsis, Mathias Schlögl

**Affiliations:** 1 Department of Musculoskeletal Biology, Institute of Life Course and Medical Sciences, University of Liverpool, UK; 2 Department of Psychological Medicine, Institute of Psychiatry, Psychology and Neuroscience (IoPPN), King’s College London, London, UK; 3 Geriatric Unit, Department of Internal Medicine and Geriatrics, University of Palermo, Italy; 4 Department of Physiology and Aging, University of Florida, Italy; 5 Phoenix Epidemiology & Clinical Research Branch, NIDDK Phoenix Epidemiology and Clinical Research Branch, USA; 6 Department of Information Engineering, University of Pisa, Italy; 7 Department of Agricultural, Food and Nutritional Science, University of Alberta, Canada; 8 Division of Geriatric Medicine, The University of North Carolina at Chapel Hill, USA; 9 Department for Geriatric Medicine, Klinic Barmelweid, Switzerland

**Keywords:** body composition, muscle health, Nutrition, physical activity, protein intake, sarcopenia

## Abstract

Sarcopenia, the age-related decline in muscle mass and strength is a growing challenge for older adults. This study investigates whether meeting the WHO physical activity guidelines (150–300 min/week of moderate or 75 min/week of vigorous exercise) with higher protein intake, based on the RDA, is associated with appendicular lean soft tissue index (ALSTI) and handgrip strength (HGS) in adults living in the United States aged 40–59 years. Data from the National Health and Nutrition Examination Survey 2007–2020 included 2540 adults aged ≥40 years. Physical activity (moderate/vigorous) was self-reported, while higher protein intake was assessed via two 24-hour recalls (>0.8 g/kg/bodyweight). Linear regressions, adjusted for age, sex, BMI, race, education, comorbidities, and energy-adjusted protein intake were used to assess associations with ALSTI and HGS. In fully adjusted models, vigorous activity with higher protein intake (47.1 ± 5.6 years) was associated with ALSTI only after adjustments for age, sex, BMI, race, education (*β* = 0.37, SE 0.17, *p* = 0.047; mean baseline: 8.56 ± 1.67 kg/m^2^). No associations were found in the fully adjusted model for ALSTI (*p* = 0.31), and across all models for HGS (mean baseline: 43.6 ± 10.1 kg). Moderate activity with higher protein intake was not associated with HGS or ALSTI across adjusted models. Meeting vigorous or moderate-intensity activity guidelines with higher than the RDA protein intake was not associated with ALSTI or HGS in middle-aged US adults.

## Introduction

Sarcopenia, defined as the progressive loss of skeletal muscle mass and strength, is a growing public health concern in ageing populations.^(1)^ Consensus groups, including the Global Leadership Initiative on Sarcopenia (GLIS) and the European Working Group on Sarcopenia in Older People (EWGSOP2), advocate for early identification and the implementation of preventive strategies to mitigate the socio-economic and health burdens associated with sarcopenia. Greater dietary protein intake and higher levels of physical activity are key modifiable factors that support muscle health and have been proposed both for its prevention and its treatment. Studies have shown that protein intakes exceeding the RDA (0.8 g/kg/ideal body weight) are associated with greater appendicular lean soft tissue (ALST), reflective of muscle mass.^(2, 3)^ Yet, most US adults do not meet these thresholds.

Resistance exercise is the most effective stimulus for muscle protein synthesis,^(4)^ particularly when combined with a high-protein diet.^(5)^ Research has also shown that aerobic exercise has a significant impact on improving muscle structure and hypertrophy in older adults, together with improved cardiorespiratory fitness.^(6–8)^ While intervention studies support the synergistic effects of protein supplementation and resistance exercise on muscle outcomes, there is growing interest in whether similar benefits may be observed in community settings, particularly when individuals meet the WHO’s recommended aerobic physical activity guidelines (150–300 min/week moderate or 75 min/week vigorous activity), along with muscle-strengthening activities at least twice a week to further support skeletal muscle mass and function.^(9)^

The fifth decade of life represents a critical window for the prevention of sarcopenia, as this period coincides with the onset of decline in muscle quality and neuromuscular integrity.^(10)^ Anabolic resistance, which is defined as the impaired muscle responses to anabolic stimuli, has been shown to become more pronounced with age, highlighting the need for early intervention.^(11, 12)^ ALST and handgrip strength (HGS) have been identified as reliable markers of muscle mass and upper body strength, respectively. These are linked to frailty, mobility, and mortality risk.^(13,14)^ However, epidemiological evidence about the impact of vigorous physical activity and protein intake on outcomes typical of sarcopenia is still limited. This study aimed to investigate whether meeting WHO physical activity guidelines, particularly vigorous activity, combined with higher protein intake, is associated with greater ALST and HGS in US adults aged 40–59 years.

## Methods

**Figure 1.**
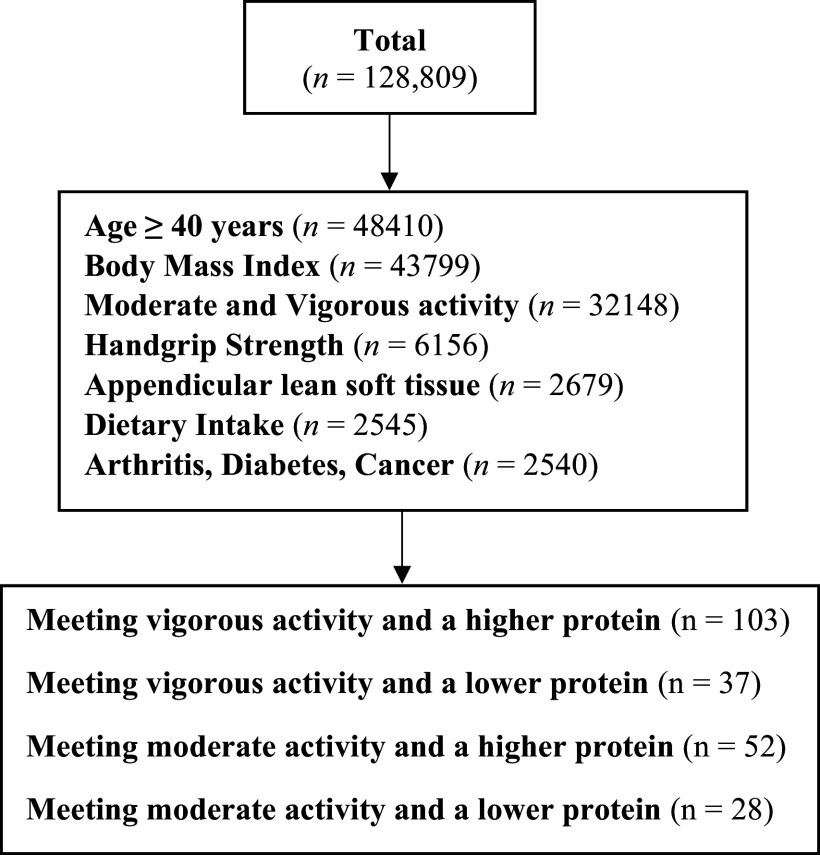
Flowchart of participant selection.

The data set was assembled from consecutive cycles of the National Health and Nutrition Examination Survey (NHANES) database, from the 2007–2008 to 2017–2020 cycles. The analysis focused on vigorous and moderate recreational activities. Furthermore, data was collected in relation to ALSTI and HGS from 2011–2012 and 2013–2014. NHANES, conducted by the National Center for Health Statistics (NCHS) of the US Centers for Disease Control and Prevention (CDC), is a series of cross-sectional surveys that collect data through interviews, physical exams, and laboratory tests. The NHANES dataset is publicly accessible through the NCHS website (https://www.cdc.gov/nchs/nhanes/index.html). The study was conducted in accordance with the ethical guidelines established by the Declaration of Helsinki and adhered to the standards set out in the STROBE Statement for reporting observational research.^(15)^ The research protocol was reviewed and approved by the NCHS Ethics Review Committee, and all participants provided written informed consent prior to enrolment. Previous studies have outlined the recruitment and study design.^(16)^ A flowchart illustrating the selected sample size is presented in Figure 1.

### Sociodemographic and clinical characteristics

Self-reported data on ethnicity (Mexican, non-Hispanic White, non-Hispanic Black, Hispanic, and other categories) and educational attainment (college degree or higher, college or associate degree, high school diploma, or other) were collected. Additionally, health conditions including arthritis, cancer, and diabetes were self-reported using the question: ‘Have you ever been told by a doctor or other health professional that you had cancer or a malignancy of any kind/diabetes/arthritis?’. BMI was calculated by dividing the participant’s body weight by the square of their height. At the Mobile Examination Center, blood samples were collected during physical exams to measure serum albumin, total cholesterol, and HDL-cholesterol (HDL-C). The samples were drawn into tubes containing ethylene diamine tetraacetic acid to prevent clotting. Following centrifugation to isolate the serum, the samples were cryogenically stored at approximately −70°C and transported to a designated laboratory for analysis. Serum albumin levels were measured using the DxC800 with the bichromatic digital endpoint method, where albumin binds with Bromcresol Purple dye. The evaluation of total cholesterol and HDL-C concentrations were conducted through enzymatic techniques.

### Physical activity

Physical activity levels were assessed through the NHANES Physical Activity Questionnaire, which records self-reported participation in moderate and vigorous recreational activities. For instance, the question ‘How much time do you spend doing vigorous/moderate-intensity sports, fitness or recreational activities on a typical day?’ was posed. The outcomes were presented in minutes per day. Participants were considered to be meeting physical activity guidelines if they reported engaging in at least 150 minutes per week of moderate-intensity activity or 75 minutes per week of vigorous-intensity activity,^(17)^ aligning with WHO standards. The weekly totals were calculated by multiplying the daily minutes by seven.

### Protein intake

Protein consumption was measured using two 24-hour dietary recalls conducted by interviewers. The average daily protein intake over the two-day period was calculated in grams per day (g/day). The mean intake was then adjusted for bodyweight (in grams per kilogram per day). The RDA of 0.8 g/kg/bodyweight was used to define ‘higher’ and ‘lower’ groups. Nutrient estimates were calculated using the United States Department of Agriculture’s Food and Nutrient Database for Dietary Studies.^(18)^

### Handgrip strength and appendicular skeletal muscle index

A Takei Digital Grip Strength Dynamometer (Model T.K.K.5401; Takei Medical) was employed for the measurement of HGS (kg). HGS was performed through three attempts for each arm, combining the maximum value from attempt into a single value. Participants performed were allowed to have 60 s of rest between each attempt. The combination of the maximum levels of strength from both arms was then divided by two, to obtain an average value.

ALST was estimated using dual-energy X-ray absorptiometry (DXA) and calculated as the sum of the lean soft tissue (kg) in the upper and lower limbs (excluding bone mineral content) divided by height squared (m^2^), terms ALST index or ALSTI as per the recent methodological standards for body composition.^(19)^ The whole-body scans were acquired on the Hologic Discovery model A densitometers (Hologic, Inc., Bedford, Massachusetts), using software version Apex 3.2, and were administered by trained and certified radiology technologists.

### Statistical analysis

The normality of continuous variables was assessed using the Shapiro–Wilk test. Baseline characteristics were compared using independent *t*-tests or Mann–Whitney *U* tests for continuous variables, and chi-squared test for categorical variables. Linear regression models were used to examine the association between combinations of physical activity levels (moderate or vigorous) and protein intake (higher or lower) with ALSTI and HGS, both treated as continuous outcomes. Physical activity and protein intake were treated as categorical variables. Weekly activity was computed by multiplying daily minutes of moderate or vigorous by seven, and participants were classified according to whether they met WHO-recommended thresholds. To ensure the results accurately represent the US population, we adhered to the sampling design and weighting methodology delineated in the NHANES analytical guidelines. The NHANES employs a multistage probability sampling framework, incorporating strata, primary sampling units (PSUs), and clusters to construct a nationally representative sample. Sampling weights were applied to address differential selection probabilities, nonresponse, and oversampling of specific demographic subgroups, such as racial minorities, older adults, or low-income individuals. Furthermore, appropriate strata and cluster variables were utilised to adjust for the complex survey design in variance estimation. Three models were constructed. Initially, the unadjusted model was constructed. Model 1 was adjusted for age, sex, BMI, race/ethnicity, and education. Model 2 was additionally adjusted for comorbidities including arthritis, diabetes, cancer, and energy-adjusted protein intake. To adjust for confounding by total energy intake when assessing protein intake, we applied the residual method by Willet *et al.* (1997).^(20)^ This approach accounts for variations in overall energy intake by calculating residuals from a linear regression model, with protein intake (g/day) as the dependent variable and total energy intake (kcal/day) as the independent variable. The resulting residuals, representing energy-adjusted protein intake, were used in subsequent analyses to evaluate associations in our fully adjusted model with HGS and ALSTI. This method ensures that the observed associations are independent of total energy intake, enhancing the validity of the findings. Moreover, results were expressed as *β* coefficients with corresponding SE. To account for multiple comparisons, we applied the Bonferroni correction, adjusting the significance threshold by dividing the alpha level by the number of comparisons conducted. Multicollinearity was assessed using the variance inflation factor, with values <3 indicating no significant multicollinearity. A two-sided *p* value < 0.05 was considered statistically significant. All analyses were performed SPSS version 29.0 (IBM, Armonk, NY, USA).

## Results

### Characteristics of the included sample

The sample selection process is detailed in Figure 1. Baseline characteristics stratified by physical activity levels and protein intake are reported in Table 1. Among 2540 adults aged 40 years and older with available data on HGS and ALSTI, 80 (3.1%) and 140 (5.5%) individuals met the WHO’s guidelines for moderate and vigorous physical activity, respectively. Of those classified as having vigorous activity, 103 (73.6%) reported a protein intake above the RDA. A similar trend was observed in the participants who met moderate activity guidelines, with 52 (65.0%) reporting a higher protein intake. Among individuals meeting the vigorous activity guidelines, those with greater protein intake were significantly younger, had lower BMI, and reported a lower prevalence of arthritis compared to those with lower protein intake. No significant differences were observed in sex distribution, race, education, or comorbidities such as diabetes or cancer. In the moderate activity subgroup, individuals with higher protein intake exhibited significantly lower BMI, as well as lower weekly minutes of moderate activity, and lower ALSTI, compared to those with lower protein intake. Differences in racial and educational distribution were also reported, while other demographic and clinical variables remained comparable between groups.

**Table 1.** Baseline characteristics based on physical activity and protein intake levels. Data are expressed as mean and standard deviation Table 1 long description.

Outcomes	Meeting vigorous activity & higher protein	Meeting vigorous activity & lower protein	*p*-Value	Meeting moderate activity & higher protein	Meeting moderate activity & lower protein	*p*-Value
Sample size (M/F)	103 (82/21)	37 (26/11)	0.26	52 (40/12)	28 (18/10)	0.30
Age (years)	47.1 (5.6)	49.4 (5.2)	0.03^*^	49.2 (6.1)	49.9 (5.8)	0.60
Race *n* – (%)						
Mexican American	23 (22.3)	4 (10.8)	–	14 (26.9)	0 (0)	–
Other Hispanic	28 (27.2)	14 (37.8)	0.24	8 (15.4)	8 (28.6)	0.03^*^
Non-Hispanic White	31 (30.1)	15 (40.5)	–	18 (34.6)	14 (50)	–
Non-Hispanic Black	9 (8.7)	1 (2.7)	–	4 (7.7)	3 (10.7)	–
Other race	12 (11.7)	6 (16.2)	–	8 (15.4)	3 (10.7)	–
Education *n* – (%)						
College or above	43 (41.7)	13 (35.1)	–	21 (40.4)	5 (17.9)	–
College or associate degree	31 (30.1)	9 (24.3)	0.65	12 (23.1)	7 (25)	0.04^*^
High school	17 (16.5)	9 (24.3)	–	7 (13.5)	8 (28.6)	–
Other (below high school)	12 (11.7)	6 (16.2)	–	12 (23.1)	8 (28.6)	–
BMI (kg/m^2^)	26.9 (4.3)	28.9 (5.7)	0.02^*^	27.8 (5.6)	33.0 (6.5)	<0.01^*^
Bodyweight (kg)	80.4 (15.3)	86.1 (18.5)	0.07	80.4 (18.6)	95.6 (20.8)	<0.01^*^
Arthritis *n* – (%)	9 (8.7)	9 (24.3)	0.02^*^	10 (19.2)	6 (21.4)	1.00
Cancer *n* – (%)	1 (1)	2 (5.4)	0.17	3 (5.8)	4 (14.3)	0.23
Diabetes *n* – (%)	6 (5.8)	2 (5.4)	0.96	6 (11.5)	4 (14.3)	0.46
Vigorous activity (min/week)	138.3 (76.4)	144.7 (57.3)	0.64	32.7 (63.7)	25.5 (65.8)	0.64
Meeting vigorous activity guidelines (%)	4.1	1.5	–	–	–	–
Moderate activity (min/week)	54.4 (63.4)	75.5 (101.0)	0.14	225.2 (48.6)	261.4 (99.6)	0.03^*^
Meeting moderate activity guidelines (%)	–	–	–	2.0	1.1	–
Sedentary activity (min/day)	383.7 (208.5)	368.1 (228.1)	0.70	403.0 (193.3)	340.7 (174.4)	0.16
Energy intake (kcal/day)	2531 (980)	1467 (683)	<0.01^*^	2458 (731)	1550 (666)	<0.01^*^
Protein intake (g/day)	108.4 (38.0)	50.9 (17.3)	<0.01^*^	101.6 (30.7)	53.6 (24.3)	<0.01^*^
Protein intake (g/kg/bodyweight)	1.36 (0.46)	0.59 (0.15)	<0.01^*^	1.31 (0.53)	0.56 (0.19)	<0.01^*^
Carbohydrate intake (g/day)	306.7 (142.6)	188.6 (98.3)	<0.01^*^	291.4 (102.6)	180.8 (82.3)	<0.01^*^
Fibre intake (g/day)	23.3 (10.1)	12.8 (6.5)	<0.01^*^	20.0 (8.8)	12.4 (7.9)	<0.01^*^
Fat intake (g/day)	95.1 (45.7)	51.8 (29.6)	<0.01^*^	96.5 (38.4)	59.1 (32.9)	<0.01^*^
Total cholesterol (mg/dL)	186.8 (56.4)	189.7 (61.4)	0.79	194.8 (45.9)	181.4 (61.7)	0.28
HDL-cholesterol (mg/dL)	50.0 (17.9)	51.1 (23.7)	0.39	51.2 (16.3)	46.1 (18.2)	0.21
Hb (mg/L)	13.9 (3.1)	13.6 (3.6)	0.30	14.1 (2.6)	13.7 (3.2)	0.58
Serum albumin (g/L)	4.13 (0.98)	3.95 (1.22)	0.19	4.21 (0.68)	3.82 (1.14)	0.06
Handgrip strength (kg)	43.6 (10.1)	42.2 (9.7)	0.46	41.7 (9.9)	40.5 (10.3)	0.61
Appendicular lean soft tissue index (kg/m^2^)	8.56 (1.67)	8.62 (1.69)	0.87	8.26 (1.34)	9.00 (1.39)	0.02^*^

* Indicates significance at *p* <0.05.

### Associations of physical activity and protein intake with muscle strength and mass

Associations between physical activity, protein intake, and muscle outcomes are presented in Table 2. Among adults meeting vigorous physical activity recommendations, higher protein intake was not associated with HGS in any of the models (Unadjusted: *β* = 1.96, SE 2.12, *p* = 0.37; Model 1: *β* = −0.22, SE 1.45, *p* = 0.88; Model 2: *β* = −0.31, SE 1.57, *p* = 0.84). On the contrary, although higher protein intake was not significantly associated with ALSTI in the unadjusted model (*p* = 0.78), a positive association was observed in Model 1 (*β* = 0.37, SE 0.17, *p* = 0.047), which was attenuated and no longer significant in Model 2 (*β* = 0.19, SE 0.18, *p* = 0.31). Following Bonferroni correction for multiple comparisons, Model 1 was not deemed significant, considering the *p* value > 0.017.

**Table 2.** Association of meeting vigorous and moderate physical activity guidelines and higher than RDA protein intake, with handgrip strength and appendicular lean soft tissue index in adults 40–59 years of age Table 2 long description.

Meeting vigorous physical activity guidelines									
	Unadjusted			Model 1			Model 2		
Outcomes	*β*	SE	*p*	*β*	SE	*p*	*β*	SE	*p*
Handgrip strength (kg)	1.96	2.12	0.37	−0.22	1.45	0.88	−0.31	1.57	0.84
Appendicular lean soft tissue index (kg/m^2^)	0.13	0.46	0.78	0.37	0.17	0.047^*^	0.19	0.18	0.31

Meeting moderate physical activity guidelines									
	Unadjusted			Model 1			Model 2		
Outcomes	*β*	SE	*p*	*β*	SE	*p*	*β*	SE	*p*
Handgrip strength (kg)	0.97	2.82	0.73	2.39	2.07	0.27	2.92	2.02	0.17
Appendicular lean soft tissue index (kg/m^2^)	−0.93	0.34	0.01^*^	−0.21	0.18	0.26	−0.11	0.15	0.47

Model 1: adjusted for age, sex, BMI, race, and education.

Model 2: adjusted for Model 1 and arthritis, cancer, diabetes, and energy-adjusted protein intake.

* Indicates significance at *p* <0.05.

In adults meeting moderate physical activity guidelines, higher protein intake was not associated with HGS (Unadjusted: *β* = 0.97, SE 2.82, *p* = 0.73; Model 1: *β* = 2.39, SE 2.07, *p* = 0.27; Model 2: *β* = 2.92, SE 2.02, *p* = 0.17). For ALSTI, an inverse association was observed in the unadjusted model (*β* = −0.93, SE 0.34, *p* = 0.01), but this effect was attenuated and became non-significant after adjustments (Model 1: *β* = −0.21, SE 0.18, *p* = 0.26; Model 2: *β* = −0.11, SE 0.15, *p* = 0.47).

## Discussion

This study found that, among US adults aged 40 years or older, neither vigorous nor moderate physical activity combined with higher protein intake was consistently associated with greater ALSTI or HGS. A modest association with ALSTI was observed in partially adjusted models for vigorous activity, but this did not remain significant after full adjustment and correction for multiple testing. In the moderate activity subgroup, an unexpected lower ALSTI was observed in those with higher protein intake at baseline, although this difference was not confirmed in adjusted models. Overall, our findings suggest that meeting WHO physical activity guidelines, even at vigorous intensity, may not be sufficient to improve muscle mass or strength when combined with protein intake, particularly in the absence of detailed information on the type of activity performed. These results differ from our initial hypothesis and highlight the complexity of the relationship between diet, physical activity, and muscle outcomes. While prior studies have highlighted the potentially synergistic effects of protein supplementation and exercise, our data indicate that without distinguishing between aerobic and resistance activities, no robust benefits on lean mass or strength can be demonstrated. This observation is consistent with experimental evidence that resistance exercise remains the most effective driver of muscle protein synthesis and hypertrophy, especially when paired with adequate protein intake.^(21,22)^ Evidence on the relationship between protein intake and HGS remains unclear. A recent systematic review and meta-analysis concluded that higher protein intake has uncertain effects on HGS, with benefits that are marginal or not significant in the absence of resistance exercise.^(23)^ In our study, we similarly did not observe significant associations. Given that HGS primarily reflects upper limb strength, it may be less sensitive to the general categories of moderate or vigorous activity captured by NHANES and more responsive to resistance-type training. Beyond muscle mass and strength, muscle power is emerging as a sensitive and clinically relevant marker of early decline in physical function, being strongly associated with higher risk of mobility loss, functional dependence, falls, and other adverse outcomes.^(24, 25)^

Several methodological considerations, however, should be taken into account when interpreting these findings. Our dichotomous classification of protein intake, based on the RDA of 0.8 g/kg/day, may underestimate the non-linear, dose–response relationships suggested by previous meta-analyses, where benefits plateau around 1.6 g/kg/day.^(26)^ Moreover, the NHANES questionnaire does not differentiate between aerobic from resistance activity, limiting the interpretation of our results. This distinction is crucial, since resistance training is most directly linked to improvements in muscle mass and strength, while aerobic exercise primarily supports cardiorespiratory fitness and muscle quality. Several individual factors, including age, comorbidities, sex differences, and dietary patterns, may modify responses to protein intake and physical activity. Women, for example, often engage in less structured exercise than men but may experience greater relative benefits in cardiovascular outcomes,^(27)^ raising the question of whether similar sex-specific effects extend to musculoskeletal ageing. Insulin resistance may also be relevant in this context, as impaired insulin signalling can influence skeletal muscle protein turnover and the anabolic response to dietary protein and exercise. In our analyses, BMI and self-reported diabetes were included as clinical covariates, but these variables do not directly capture insulin sensitivity. Because of the cross-sectional design, we could not reliably assess whether insulin resistance acted as a mediator, confounder, or downstream correlate of differences in protein intake and skeletal muscle outcomes. Reverse causality also remains possible, as individuals with better muscle health may be more likely to maintain both higher protein intake and greater physical activity levels. Finally, both dietary intake and physical activity were self-reported, introducing recall and social desirability bias. Specifically, the marked differences in reported total energy intake between higher and lower protein groups should be interpreted with caution. Participants in the lower protein groups also reported lower total energy and macronutrient intake, which may reflect true dietary differences but also potential underreporting. Although two 24-hour recalls and energy-adjusted protein intake were used to reduce this bias, residual dietary misclassification cannot be excluded.

## Conclusions

In this nationally representative sample of US adults aged 40 years or older, meeting vigorous or moderate physical activity guidelines in combination with higher protein intake was not consistently associated with greater ALSTI or HGS. A modest association observed in partially adjusted models did not persist after full adjustment and correction for multiple testing. These null findings warrant caution when extrapolating synergistic effects of protein intake and physical activity in midlife. Longitudinal and interventional studies are needed to clarify whether resistance training, combined with adequate protein intake, yields measurable benefits. Future research should also explore sex-specific responses, protein quality, and additional muscle-related metrics, including muscle power.

## Table 1. Long description

The table presents baseline characteristics based on physical activity and protein intake levels. It has 25 rows and 11 columns. The columns are labeled as Outcomes, Meeting vigorous activity & higher protein, Meeting vigorous activity & lower protein, p-Value, Meeting moderate activity & higher protein, Meeting moderate activity & lower protein, and p-Value. The rows include various outcomes such as Sample size (M/F), Age (years), Race, Education, BMI (kg/m^2), Bodyweight (kg), Arthritis, Cancer, Diabetes, Vigorous activity (min/week), Meeting vigorous activity guidelines, Moderate activity (min/week), Meeting moderate activity guidelines, Sedentary activity (min/day), Energy intake (kcal/day), Protein intake (g/day), Protein intake (g/kg/bodyweight), Carbohydrate intake (g/day), Fibre intake (g/day), Fat intake (g/day), Total cholesterol (mg/dL), HDL-cholesterol (mg/dL), Hb (mg/L), Serum albumin (g/L), Handgrip strength (kg), Appendicular lean soft tissue index (kg/m^2). Each cell contains specific values or percentages related to the outcomes and categories.

Navigate back to Table 1..

## Table 2. Long description

The table presents data on the associations between physical activity, protein intake, and muscle outcomes in adults aged 40-59 years. It is divided into two main sections: meeting vigorous physical activity guidelines and meeting moderate physical activity guidelines. Each section includes three columns: Unadjusted, Model 1, and Model 2, with rows for Handgrip strength (kg) and Appendicular lean soft tissue index (kg/m^2). The columns display beta coefficients (β), standard errors (SE), and p-values (p). For vigorous physical activity, higher protein intake was not associated with handgrip strength in any model. For appendicular lean soft tissue index, a positive association was observed in Model 1 but was attenuated in Model 2. For moderate physical activity, higher protein intake was not significantly associated with handgrip strength in any model. For appendicular lean soft tissue index, a negative association was observed in the unadjusted model but was not significant in Models 1 and 2.

Navigate back to Table 2..
